# Integromics profiling of oral carcinoma: exploring the role of miRNAs and circRNAs

**DOI:** 10.3389/froh.2025.1682713

**Published:** 2025-10-23

**Authors:** Ioannis Alexandros Charitos, Gilberto Sammartino, Sandro Rengo, Salvatore Scacco, Marco Tatullo

**Affiliations:** 1 Department of Translational Biomedicine and Neuroscience—DiBraiN, University of Bari “Aldo Moro”, Bari, Italy; 2 Department of Neurosciences, Reproductive and Odontostomatological Sciences, University of Naples “Federico II”, Naples, Italy

**Keywords:** oral cancer, oral squamous cell carcinoma, biochemistry, non-coding RNAs, cancer-related omics technology, integromics, pan-omics

## Abstract

Oral squamous cell carcinoma (OSCC) remains a formidable challenge in modern medicine, threatening enormous number of lives worldwide. Although research is offering an exponential growth as numbers of molecular pathways, biomarkers, and potential therapeutic targets involved in cancer onset and development, the major bottleneck is represented by the identification and characterization of novel theranostic compounds. Recently developed integrative omics (referred as integromics or pan-omics) methodology is offering promising angles in this field by combining diverse datasets, such as genomic, epigenomics, transcriptomic, proteomic, lipidomics and metabolomic, with computational models and experimental findings, highly demanded for a deeper insight into the molecular mechanisms underlying cancer progression, treatment responses and resistance. In this context, non-coding RNAs (ncRNAs), such as microRNAs (miRNAs) and circular RNAs (circRNAs) can be used as targets in OSCC. Thus, these genomic techniques focus on enriching fragments related to protein-coding genes and specific regulatory RNAs, such as microRNAs. By integrating mutational databases, patient genomic and clinical data, and therapeutic action databases, this approach improves both primary and secondary prevention of cancer. Specifically, it enhances preventive effectiveness by identifying which somatic mutations in a patient's tumor can be targeted with specific therapies. MiRNAs and circRNAs, whose dysregulation is particularly evident in several stages of tumorigenesis, including metastasis and immunosuppression, alongside treatment resistance, function as regulators of gene expression. Thus, integromic studies are nowadays investigating their involvement as diagnostic biomarkers for early detection and prognosis, ultimately facilitating precision and personalized oncology, with significant improvement of patient outcomes. Additionally, the integration of advanced imaging technologies and targeted therapies, referred as theranostic, is revolutionizing the field of oncology in all its facets. Such approach improves therapy effectiveness by tackling specific characteristics, while simultaneously monitoring patient's response.

## 1 Introduction

Oral squamous cell carcinoma (OSCC), a leading subtype of head and neck cancer, is the seventh most common cancer in male under the age of 60, with high global incidence and mortality rate (1). According to the World Health Organization (WHO), head and neck cancers are collectively the sixth most common cancer worldwide, with squamous cell carcinoma being the most frequent type. Specifically, oral cancer alone accounts for approximately 390,000 new cases and 190,000 deaths globally each year, making it the 16th most diagnosed cancer and the 15th leading cause of cancer death (2). Despite recent progress in understanding the complex molecular changes leading to OSCC development, the etiology remains largely unknown, encompassing genetic and environmental factors (3). While many molecular mechanisms involved in OSCC have been identified, the complete, unified picture of how these events integrate over time to drive carcinogenesis is still lacking. Molecular integromic, often bridged to computational methods, is a relatively recent interdisciplinary field that amalgamates various omics platforms such as genomics, epigenomics, transcriptomics, proteomics, lipidomics and metabolomics, providing a holistic understanding of the dynamics of biological systems in the context of complex conditions, including those belonging to cancer heterogeneity and treatment response (4). This approach has generated a vast number of targeting biomarker candidates to tackle cancer onset and progression. Additionally, by incorporating computational models and artificial intelligence (AI), integromic is revealing unexpected molecular interactions within the tumor microenvironment (TME), paving the way for novel synergistic drug combinations overlooked using traditional methods (5). Among others, the integromic approach has greatly advanced our understanding on the role of non-coding RNAs (ncRNAs), such as microRNAs (miRNAs) and circular RNAs (circRNAs) in cancer onset (6, 7). Long considered transcriptional “noise”, these two classes of RNA are now recognized as crucial intermediate of various cellular processes, including cell proliferation, migration and apoptosis. Functionally, miRNAs, as well as circRNAs, supervises gene expression at post-transcriptional level, altering signaling pathways critical for cancer development and progression (8). Initially detected in *Caenorhabditis elegans* (*C. elegans*) in 1993, miRNAs are synthesized in the nucleus as about 1,000 nucleotides in length, processed in hairpin structures as pre-miRNA of roughly 80 nucleotides and carried to the cytoplasm where they are finally resized into their mature form consisting of∼18–25 nucleotides. By binding to the 3′ untranslated regions (UTRs) of target messenger RNAs (mRNAs) through a multistep mechanism mediated by the RNA-induced silencing complex (RISC), miRNAs guide transcript degradation and gene silencing (9). CircRNAs were first identified in the murine respirovirus (*Sendai virus*) and in a species of fruit fly (*Ceratitis capitata*) in the 1970s. Represented as single-stranded RNAs, circRNAs are characterized by their covalently closed loop structure. A peculiar form of splicing, referred as back-splicing, occurs in the case of circRNAs, and involves the joining of a downstream 5′ splice site to an upstream 3′ splice site, resulting in the circularization of the RNA (10). Through integromic approaches, researchers have pinpointed several aberrantly expressed miRNAs and circRNAs, contributing to different cancer stages, metastatization or resistance to therapies (11). Newly identified RNAs are constantly incorporated into growing integrative maps of regulatory elements, including upstream and downstream regulators and targets, which uncover, ultimately, new networks for diagnostic, prognostic and/or predictive biomarker recognition (12). Besides, miRNAs and circRNAs emerge as potential therapeutic agents in different type of cancers, offering new opportunities in cancer management. Up-to-date technologies are in fact realizing functional engineered nanostructured carriers encapsulating miRNAs and circRNAs aimed to specifically destroy cancer cells, without affecting healthy surrounding tissues (13). The codevelopment of diagnostic and therapeutic tools, known as theranostic, is a multidisciplinary approach nowadays employed to ease patient care and to tailor effective treatment strategies against cancer (14). By empowering imaging probes and techniques, theranostic enables cancer detection at the molecular or even cellular level, long before clinical symptoms arise, thus holding the potential to revolutionize malignancies in their early stages (14). Recently, scientific progress on theranostic strategies let the identification of novel genetic mutations or metabolic alterations within the neoplastic cells, crucial to dictate the appropriate therapeutic regimens (15). As an evolving field catering solutions to the unmet needs in oncology, one of the most promising areas in cancer theranostic is the development of nanocarriers designed to deliver compounds directly to cancer cells, while simultaneously allowing for real-time tracking drug distribution throughout the body. Such approach aims to minimize toxicity to surrounding healthy tissues by reducing off-target effects (16). Thus, integromic applied to cancer theranostic has opened new avenues for understanding tumor biology and developing more personalized targeted therapies. Aim of this review is to firstly summarize the knowledge on the integromic approaches currently employed to identify novel candidates in cancer treatment. Additionally, we will examine how multi-omics profiling leads to the identification of novel potential candidates underpinning OSCC development. Finally, we will dissect the potential use of miRNAs and circRNAs as therapeutic agents.

## 2 From genes to proteins: A molecular holistic overview in cancer pathophysiology

Molecular reprogramming driving cancer heterogeneity encompasses genomic instability, gene expression changes, mutations, epigenetic and post-translational modifications, altered regulation of oncogenes and tumor suppressors, and aberrant protein function. These changes lead to widespread genomic, transcriptomic, and proteomic rewiring. Such molecular perturbations can impact critical cellular pathways, including cell cycle control, apoptosis, angiogenesis, and metastasis. Therefore, integrative multi-omics approaches are becoming increasingly essential for dissecting this layered heterogeneity, offering insights into cancer pathophysiology and informing precision oncology strategies. In the following subsections, we describe recent advances in these technologies and their applications in tumor biology.

### 2.1 Recent advances in omics technology

**Figure 1:**
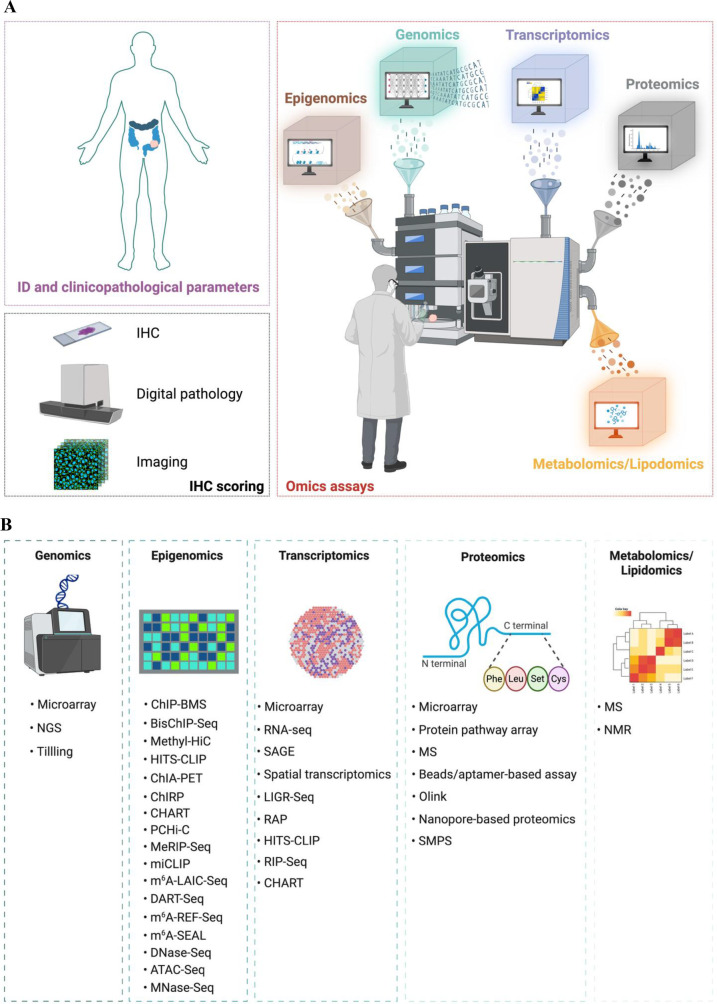
Omics-based approaches employed for the molecular characterization of cancer. **(A)** A comprehensive understanding of individual's cancer properties is required for the adequate treatment decisions, with multidimensional perspective for personalized treatment plans. The integration of clinicopathological parameters with the presence of specific antigens (detected through immunohistochemistry, digital pathology and imaging techniques) and defined molecular profiles (derived from genomics, epigenomics, transcriptomics, proteomics, metabolomics and lipidomics assays) enables more accurate diagnoses, which ultimately improves patient outcomes. **(B)** genomics technology encompasses microarrays, next-generation techniques (NGS) and target induced local lesions in genomes (TILLING); epigenomics studies are performed via several methods embracing ChIP-bisulfite methylation sequencing (ChIP-BMS), bisulfite-treated ChIP DNA-sequencing (BisChIP-Seq), Methyl-High-throughput chromosome conformation capture (Methyl-HiC), Chromatin interaction analysis with paired-end tag sequencing (ChIA-PET), Chromatin isolation by RNA purification (ChIRP), promoter capture Hi-C (PCHi-C), m^6^A/methylated RNA immunoprecipitation sequencing (MeRIP-Seq), m^6^A individual-nucleotide resolution UV crosslinking and immunoprecipitation (miCLIP), m^6^A-level and isoform-characterization sequencing (m^6^A-LAIC-Seq), deamination adjacent to RNA modification targets (DART-Seq), m^6^A-sensitive RNA-Endoribonuclease–Facilitated sequencing or m^6^A-REF-sequencing (m^6^A-REF-Seq), m^6^A-selective chemical labeling (m^6^A-SEAL), deoxyribonuclease I (DNase I) hypersensitive site sequencing (DNase-Seq), Assay for Transposase-Accessible Chromatin using sequencing (ATAC-Seq), and micrococcal nuclease sequencing (MNase-Seq). Conventional transcriptomics methods include microarrays, RNA sequencing (RNA-Seq), Serial Analysis of Gene Expression (SAGE), spatial transcriptomics, high-throughput sequencing of RNA isolated by crosslinking immunoprecipitation (HITS-CLIP), ligation of interacting RNA followed by high-throughput sequencing (LIGR-Seq), Capture Hybridization Analysis of RNA Targets (CHART), RNA immunoprecipitation sequencing (RIP-Seq), and RNA antisense purification (RAP). Cutting-edge proteomics techniques embrace mass spectrometry (MS), protein pathway array, next generation tissue microarrays, multiplex bead- or aptamer-based assays, proximity extension assay (Olink), nanopore based single-molecule proteomics, and single molecule protein sequencing (SMPS). MS and nuclear magnetic resonance (NMR) are engaged in metabolomics and lipidomics assays.

Cancer is a complex disease that arises from the interaction of multiple molecular pathways engaged in intricate networks rarely operating in isolation (17). Several omics techniques, summarized in Figure 1A, have been developed to decipher the complexity of the pathology, spanning from genes and RNAs, to proteins and metabolites, which complement results obtained from immunohistochemistry analyses and clinicopathological datasets. Historically considered the progenitor of the various omics, genomics focuses on the structure, function, evolution, mapping, and editing of entire genomes to dissect associations between human diseases and genetic alterations (18). The two major technologies for genomic studies are next-generation sequencing (NGS)-based tests: the whole-genome sequencing (WGS) and whole-exome sequencing (WES). Moreover, array-based hybridization (microarrays) and target induced local lesions in genomes (TILLING) are tools employed in genomic studies (19, 20). Besides cancer-related genomics changes, epigenetic alterations perform crucial roles in cancer onset and development. Epigenomics interrogates the physical modifications across an individual's entire genome, including DNA methylation and histone modification, which can influence gene expression without altering the genetic code, as well as chromatin accessibility and chromosome conformation (21). Several methods have been engineered to investigate the methylation of the DNA, such as DNA ChIP-BMS (chromatin immunoprecipitation—bisulfite methylation sequencing) and bisulfite-treated ChIP DNA-sequencing (BisChIP-Seq). Chromosome conformation capture (3C), including Methyl-High-throughput chromosome conformation capture (Methyl-HiC), 4C, 5C, Chromatin interaction analysis with paired-end tag sequencing (ChIA-PET), Chromatin isolation by RNA purification (ChIRP), and promoter capture Hi-C (PCHi-C) are methods employed when studying chromosome conformation and chromatin interactions. RNA modifications are examined through m^6^A/methylated RNA immunoprecipitation sequencing (MeRIP-Seq), m^6^A individual-nucleotide resolution UV crosslinking and immunoprecipitation (miCLIP), m^6^A-level and isoform-characterization sequencing (m^6^A-LAIC-Seq), deamination adjacent to RNA modification targets (DART-Seq), m^6^A-sensitive RNA-Endoribonuclease–Facilitated sequencing or m^6^A-REF-sequencing (m^6^A-REF-Seq), and m^6^A-selective chemical labeling (m^6^A-SEAL). In addition, multiple biochemical methods have been developed to analyze chromatin accessibility, such as deoxyribonuclease I (DNase I) hypersensitive site sequencing (DNase-Seq), Assay for Transposase-Accessible Chromatin using sequencing (ATAC-Seq), and micrococcal nuclease sequencing (MNase-Seq) (22, 23). Transcriptomics examines the set of all RNA transcripts, including coding and non-coding, in a single cell (SC) or a population of cells, capturing gene expression changes, instrumental in the understanding of cancer pathogenesis (24). The Bulk RNA-seq is a genetic technique that measures the average expression of genes in a population of cells or tissues, rather than individual cells. It analyzes the total RNA from many cells in a sample, yielding an “average profile” of expression. This technique is useful for comparing gene activity between different states, such as healthy and diseased states, and for detecting overall trends and differentially expressed genes (25). The single cell RNA sequencing (scRNA-seq) allows the recording of total gene expression in individual cells, providing a detailed picture of cell function and interactions in heterogeneous samples. This method analyzes the level of activity of each gene in each cell individually, which helps to identify rare cell types and dynamic states that might otherwise be missed in “bulk expression” analysis (26). Finally, spatial RNA sequencing (spRNASeq) is a technique that allows the analysis of RNA sequence in a sample, while preserving information about its spatial location, providing detailed insight into gene expression and cellular function within a tissue (24). The RNA analysis is mainly performed through microarray, high-throughput RNA sequencing (RNA-Seq), Serial Analysis of Gene Expression (SAGE), and spatial transcriptomics. NcRNAs have been investigated through high-throughput sequencing of RNA isolated by crosslinking immunoprecipitation (HITS-CLIP), RNA immunoprecipitation sequencing (RIP-Seq), RNA antisense purification (RAP), ligation of interacting RNA followed by high-throughput sequencing (LIGR-Seq), and Capture Hybridization Analysis of RNA Targets (CHART) (27, 28). Proteomics extends this understanding by investigating the interactions, function, composition, and structures of proteins and their cellular activities (29). High-throughput proteomic screenings involve mass spectrometry (MS), single molecule protein sequencing (SMPS), protein pathway array, next generation tissue microarrays, multiplex bead- or aptamer-based assays, proximity extension assay (PEA), and nanopore based single-molecule proteomics (30). Lastly, metabolomics and lipidomics examine the entire set of lipids and metabolites respectively, including associated pathways through nuclear magnetic resonance (NMR) spectroscopy or MS (31), Figure 1B. Although each of these fields add layers of information aimed to elucidate how dysregulated molecule dynamics coordinate various pathophysiological conditions, the characterization of the heterogeneity and the magnitude of the clinical and biological outcomes of certain diseases, including cancer, necessitates the integration of different omics. In addition to transcriptomics, spatial-omics can also study the proteome (spatial proteomics), the metabolome (spatial metabolomics), the lipidome (spatial lipidomics), and even the connectome (spatial connectomics), analyzing molecular distribution within tissue context. These technologies allow us to observe how different types of molecules are organized in space, providing a deeper understanding of cellular and tissue processes (32).

### 2.2 Integromics approach in tumor biology

**Figure 2:**
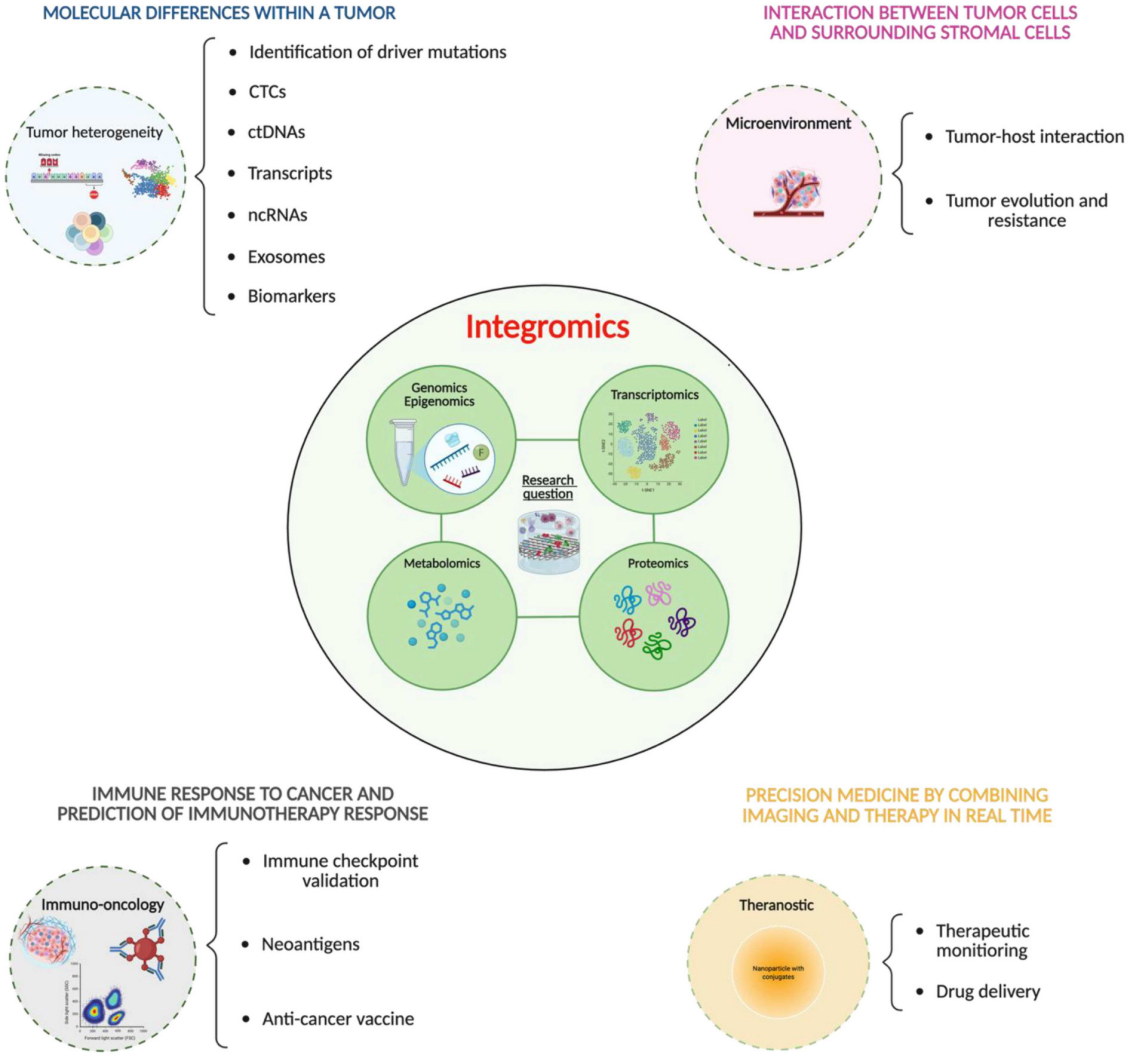
Multi-omics applications used for the molecular characterization of tumors. Based on the research questions, a multidisciplinary team employs integrative approaches to define several features of cancer, including tumor subtyping, the interaction between tumor cells and surrounding stromal cells, the immune response, and the establishment of precision medicine through the real-time combination of imaging and therapy.

Major obstacles in cancer research include tumor heterogeneity. Individuals affected by the same tumor sub-type might manifest molecular, phenotypic and clinical evolution incongruencies (33). Omics development offers novel perspectives at several stages in the oncology field, expanding the traditional tumor classification based on histotype and site of origin with genomics, epigenomics, transcriptomics, proteomics, metabolomics and lipidomics outcomes (34). However, although single-level omics approaches have enlightened several driver genes in cancer, it lacks the whole biological picture, essential to fully understand the development of the neoplasm, and ultimately, drive personalized targeted therapies, influence treatment effectiveness, and improve patient management (35). Thus, integrative approaches leveraging complementary molecular data gained from different omics are now emerging. Integromics applications could, for instance, be pivotal in establishing tumor subtyping from distinct angles, including the molecular profile and the characterization of tumor heterogeneity (36). Holistic paradigms provide, in fact, a more precise identification of driver mutations, dysregulated pathways and unconventional interactions (37). Integromics enables a more accurate interpretation of liquid biopsies by concurrently analyzing circulating tumor cells (CTCs), circulating tumor DNA (ctDNA), transcripts, non-coding RNAs (ncRNAs), exosomes, and proteins from blood (38), as well as biomarkers, including diagnostic, prognostic, and predictive markers, whose expression may reflect disease progression, recurrence, or treatment response (39). Liquid biopsies are non-invasive tests that analyze biological fluids, such as blood, to identify traces of tumors through the detection of ctDNA and other biomarkers. They are used to detect cancer, assess the effectiveness of treatment, monitor disease recurrence, and personalize treatment, especially when traditional biopsies are not feasible (40). An integrated approach has profound implications for the characterization of the tumor microenvironment (TME), providing insights into tumor–host interactions, tumor evolution and resistance, as well as the metabolic and immune backgrounds residing within the tumor niche (41). In addition, intensive research is employing integromics for the detection of novel immune checkpoints and mutation-associated antigens (neoantigens), paving the way for the development of personalized anticancer vaccines (42). Lastly, improvements in theranostic nanomedicine, which encompasses therapeutic monitoring along with drug delivery, are benefiting from a multi-omics framework (43), Figure 2.

With the advent of the immunotherapy has brought several benefits regarding patient survival (44). However, the mechanisms by which some patients do not respond to therapy remain elusive. To overcome this issue, several independent research groups are exploiting single-cell multi-omics approach to identify cell-surface markers which could serve as immune checkpoints (45). Independent multi-omics studies identify several candidates, including proteins, miRNAs, and circRNAs potentially involved in these types of cancer, nowadays, outlined in the following sections. Within the scope of multi-omics, AI has been largely used to integrate different omics layers. AI algorithms can efficiently integrate and classify heterogenous data allowing early cancer detection and screening, tumor classification and grading or predict prognosis and treatment response (46–48). In the following sections we will dissect the recent knowledge on the use of multi-omics technology aimed at tackling the onset of OSCC from complementary angles.

## 3 Integromics profiling of OSCC

The lack of early detection and precise treatment options underpins the high morbidity and mortality rates of digestive system cancers, posing significant threats to public health. The development of multi-omics methodologies has led to an explosion of opportunities in the field of oncology. In the following sections we outline the recent multi-omics advancements that led to the discovery of dysregulated players in the onset of OSCC.

### 3.1 Integrative multi-omics analysis of OSCC

**Figure 3:**
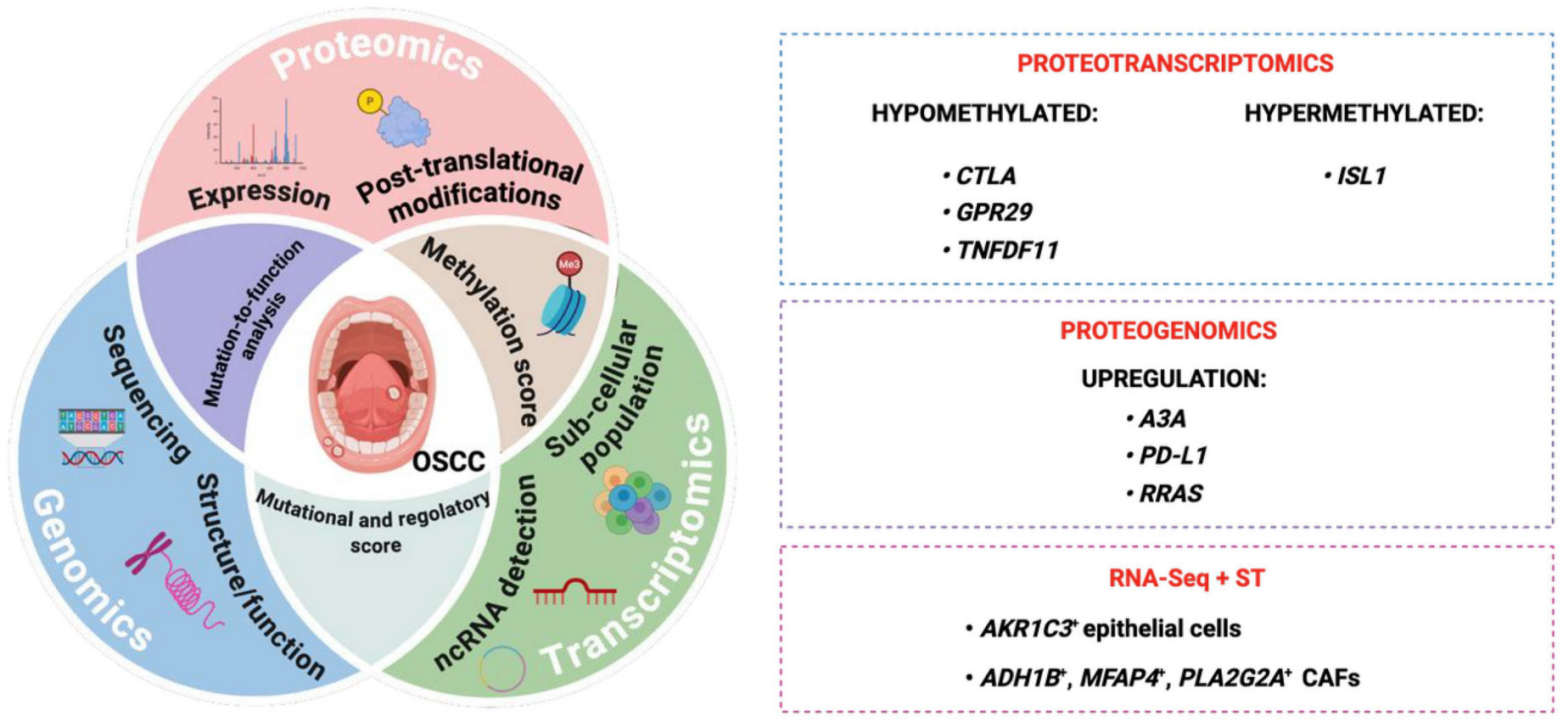
Integromics-based approach for molecular profiling in OSCC. Proteotranscriptomics analysis distinguished hypermethylated and hypomethylated proteins. Specifically, *ISL1* has been found to be hypermethylated, while *CTLA*, *GPR29*, and *TNFDF11* were hypomethylated. Proteogenomics revealed an upregulation of *A3A*, PD-L1 (related to *PDCDL1* or *CD274* gene), and RRAS (related to *RAS* gene), classified as potential biomarkers in OSCC. The combination of two transcriptomics methodologies, RNA-Seq and ST, revealed the presence of AKR1C3^+^ epithelial cells and *ADH1B^+^*, *MFAP4^+^*, and *PLA2G2A^+^* CAFs sub-populations within the tumor niche.

The molecular mechanisms underpinning the onset of OSCC are not yet fully elucidated; however, emerging studies employing omic technologies are paving the way for the development of new therapeutic options. Although integrative multi-omics approaches are still in the early stages, they display great potential against this neoplasm. Wan and colleagues uncovered an altered genotype profiling with potential clinical applications in OSCC. Preliminary RNA-seq screening identified 778 upregulated genes and 2,211 downregulated genes by comparing 17 paired samples of OSCC with the corresponding peritumoral counterpart. By integrating these findings with methylation profiling and protein-protein interaction public databases, the authors identified three hypomethylated hub genes, including Cytotoxic T-lymphocyte associated protein 4 (CTLA-4) that is both a gene and a protein (the *CTLA4* gene encodes the CTLA-4 protein), *G-protein coupled receptor 29* (*GPR29*) that encodes G protein-coupled receptor (GPCR) and *Tumor necrosis factor* (*ligand*) *superfamily member 11* (*TNFSF11*) that encodes a member of the tumor necrosis factor (TNF), and one hypermethylated hub gene, *Insulin gene enhancer protein* (*ISL1*), which may serve as novel biomarkers and therapeutic targets in OSCC (49). Recently, proteogenomics studies revealed that OSCC samples co-express high levels of Apolipoprotein B mRNA editing enzyme, *catalytic polypeptide-like 3A* (*APOBEC3A*, or *A3A*), and Programmed death-ligand 1 (PD-L1) encoded by the *CD274* gene, suggesting that *A3A* could represent an additional biomarker for identifying patients responsive to anti-PD therapy (50). Similarly, a proteogenomics analysis demonstrated that OSCC samples expressing high levels of *Rat sarcoma* (*RAS*)-related protein R-Ras (RRAS), a factor involved in several cellular processes, including proliferation, migration, and survival, are more sensitive to anti-Epidermal growth factor receptor (EGFR) targeted therapy and display more favorable clinical outcomes. Thus, this integrative, multi-layered, approach led to the identification of a novel biomarker in OSCC (50). Additionally, in 2024, Zhang and colleagues develop a comprehensive atlas of epithelial cells and cancer-associated fibroblasts (CAFs) by integrating RNA-Seq and spatial transcriptomics, and unveiling, for the first time, the complex interactions within the OSCC microenvironment. In particular, the authors detected a sub-group of epithelial cells, Aldo-keto reductase family 1 member C3 positive cells (AKR1C3^+^), located in the OSCC stroma, with high copy number variation and associated with poor prognosis. Moreover, they also identified an *Alcohol dehydrogenase 1B* (*ADH1B*)/Microfibril Associated Protein 4 (MFAP4*)/ phospholipase A2 group IIA* (*PLA2G2A*) positive CAF sub-population which interacts with AKR1C3^+^ epithelial cells, sustaining invasion (51). The MFAP4 protein is encoded by *MFAP4* gene Figure 3.

### 3.2 NcRNAs: a new frontier in the onset of OSCC

**Figure 4:**
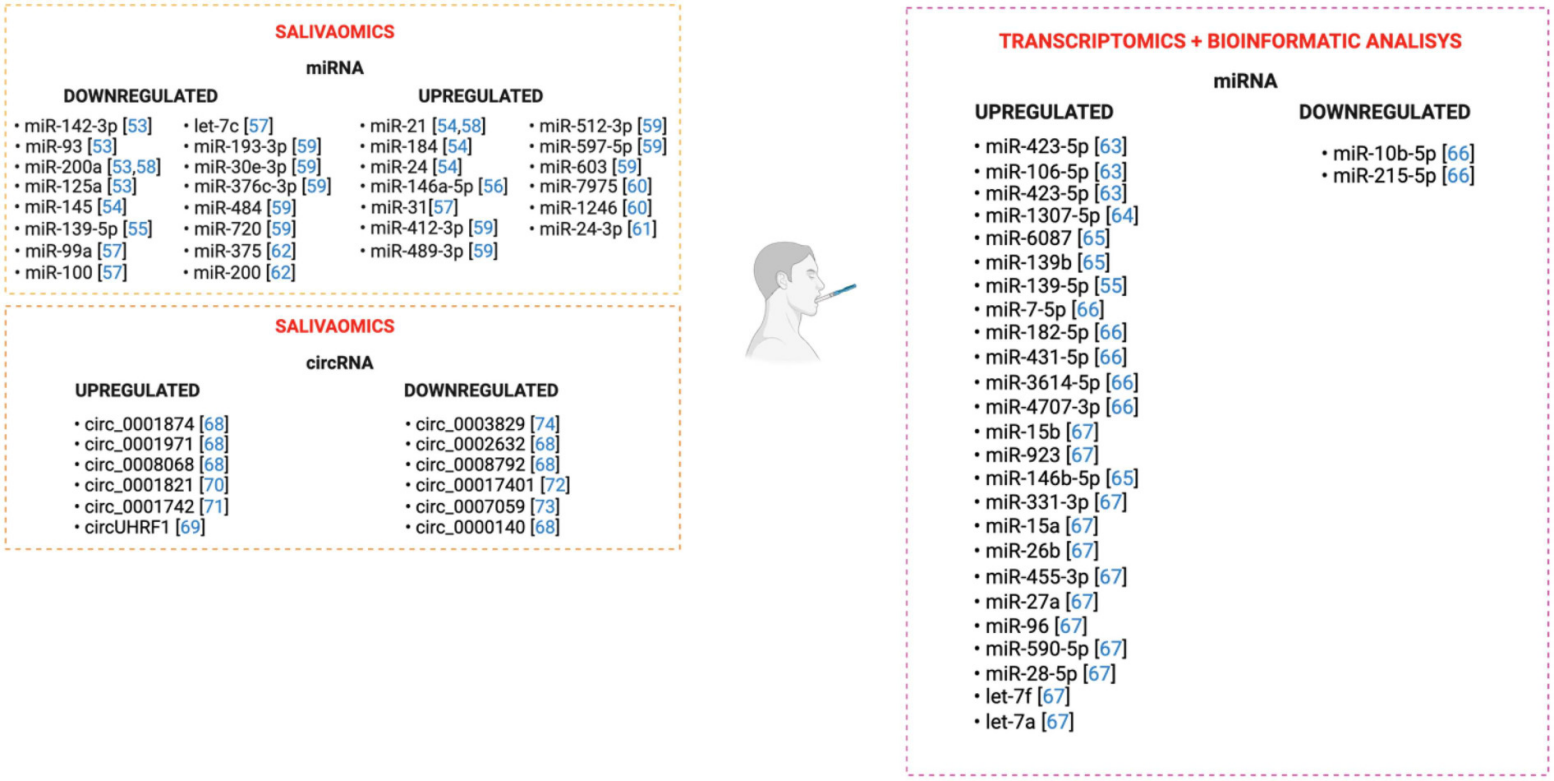
Salivaomics highlighted the aberrant expression of several ncRNAs, including miRNAs and circRNAs which may be targeted in the therapy of OSCC.

Salivaomics, the integration of various high-throughput omics methodologies, enables researchers to characterize the molecular signature and microbiota profile of salivary samples through non-invasive approach. MiRNomics analysis performed on salivary samples has allowed the detection of numerous hsa_miRNAs that could ultimately serve as potential OSCC biomarker. In particular, OSCC samples displayed a downregulation of miR-142-3p, miR-200, miR-125a, miR-93, miR-145, miR-139-5p, miR-99a, let-7c, miR-100, miR-193-3p, miR-30e-3p, miR-376c-3p, miR-484, miR-720, miR-375 and miR-200a, whereas miR-21, miR-184, miR-146a-5p, miR-24, miR-31, miR-412-3p, miR-489-3p, miR-512-3p, miR-597-5p, miR-603, miR-7975, miR-1246, miR-24-3p were upregulated compared to control tissues (52–61). Moreover, RNA-seq experiments have documented enhanced levels of miR-423-5p, miR-106-5p (62), miR-1307-5p (63), miR-6087, miR-139b (64), miR-139-5p (54), in the saliva samples of subjects diagnosed with OSCC. By integrating tumor tissue miRNA sequencing data from the Cancer Genome Atlas (TCGA) with salivary RNA-seq experiments, researchers have recognized aberrant miRNA expression in individuals with OSCC, documenting high levels of miR-7-5p, miR-182-5p, miR-431-5p, miR-3614-5p, miR-4707-3p, while miR-10b-5p and miR215-5p were found downregulated (65). Lastly, combined bioinformatic and microarrays analyses highlighted enhanced expression of several miRNAs, including miR-15b, miR-923, miR-146b-5p, miR-331-3p, miR-15a, miR-26b, miR-455-3p, miR-27a, miR-96, miR-590-5p, miR-28-5p, let-7f, and let-7a (66), Figure 4. Similarly, the function of several hsa_circRNAs has emerged through the salivaomics approach. Circ_0001874, circ_0001971, and circ_0008068 have been found overexpressed in the saliva of OSCC patients. Conversely, circ_0000140, circ_0002632, and circ_0008792 were downregulated (67). In addition, increased expression of circUHRF1 reflects enhanced levels of N-cadherin and Vimentin, while correlating with decreased *E-cadherin* (*CDH1*) expression, thus conferring invasiveness properties to OSCC cells (68). He and colleagues reported that aberrant circ_0001821 expression is involved in OSCC cell proliferation (69). Moreover, it has been shown that circ_0001742 regulates OSCC cell proliferation, epithelial-to-mesenchymal (EMT) transition and invasion (70); circ_0070401 alters the *APC2* (*Adenomatous polyposis coli protein 2* that encodes for the APC2 protein) levels (71), while circ_0007059 influences the Protein Kinase B (AKT)/mammalian target of rapamycin (mTOR) pathway, thereby affecting OSCC cell growth (72). Finally, Zhang and colleagues reported that the downregulation of circ_0003829 negatively correlated with lymph node metastasis and Tumor/Nodes/Metastasis (TNM) stage (73). Surprisingly, ncRNAs are turning out crucial drivers in the development of OSCC, enabling the optimization of innovative therapies, Figure 4.

## 4 Potential candidate diagnostic and therapeutic tools based on miRNA and circRNA

Advanced transcriptomic analyses, corroborated by functional assays, are paving the way for transcriptome-wide biomarker discovery. This has led to the identification of novel ncRNAs involved in aberrant pathways that ultimately affect OSCC physiology. By altering gene expression, miRNAs and circRNAs can either promote or suppress cancer development. Tumor-suppressor ncRNAs are often downregulated, whereas oncogenic species tend to be overexpressed, enabling their characterization for therapeutic purposes and a more nuanced understanding of OSCC development (74). In the following sections, we summarize the current knowledge about the roles of miRNAs and circRNAs in OSCC development and progression. Finally, several studies evidence have demonstrated that miRNAs and circRNAs could potentially serve as novel therapeutic tools for OSCC by modulating the basal expression.

### 4.1 The miRNA signatures for metastatic risk assessment in OSCC

With the advent of transcriptomics, a growing number of miRNAs have been implicated in pathways involved in OSCC suppression or, on the contrary, OSCC development and progression, through different mechanisms. Hui and colleagues reported that miR-101, by targeting C-X-C chemokine receptor 7 (CXCR7), which is overexpressed in OSCC, prevents its binding to C-X-C chemokine ligand 12 (CXCL12) that is encoded by the *CXCL12* gene, thus reducing cell adhesion, viability and tumor growth (75). Exosome-derived miR-34a-5p inhibits the spread of malignant cells by regulating Tyrosine-protein kinase receptor UFO (encoded by *AXL* gene) expression and modulating the EMT program (76). Recently, miR-944 has been reported to hamper cellular invasion and migration by regulating *Matrix metalloproteinase-10* (*MMP-10*) expression (77). Additionally, the EMT reprogramming in OSCC is constrained by the expression of miR-153-3p, which targets the Zinc finger protein *SNAI1* (also referred as *Snail Family Transcriptional Repressor 1*, or *Snail*) (78). Sasahira and colleagues showed that OSCC development may be also restricted upon ectopic expression of miR-126, which interferes with the vascular endothelial growth factor A (VEGF-A) that is both gene and protein, vascular endothelial growth factor C (VEGF-C) that encoded by VEGFC gene, levels (79). Recently, the panel of tumor suppressor miRNAs in the context of OSCC has been expanded to include miR-5580-3p and miR-376c-3p, which target *Laminin Subunit Gamma 2* (*LAMC2)* and *Homeobox B7* (*HOXB7*), respectively (80, 81). On the contrary, other studies based on transcriptomics assays, have identified a multitude of miRNAs that hasten OSCC progression. Notably, the expression of the tumor suppressor protein Phosphatase and tensin homolog deleted on *chromo-some 10* (*PTEN*) is regulated by several oncomiRs. miR-142-5p and miR-24 dampen PTEN expression, leading to dysregulation of the phosphatidylinositol 3-kinase (PI3K)/AKT pathway which ultimately promotes invasion, viability and chemoresistance (82, 83). It has been shown that miR-211 downregulates *bridging integrator-1* (*BIN1*) expression, enhancing OSCC proliferation, migration and invasion (84), Table 1.

**Table 1:** Involvement of different classes of miRNAs in the pathogenesis of OSCC. Transcriptomics and functional analyses provide detailed insights into the molecular mechanisms by which miRNAs either suppress or promote the pathogenesis of OSCC. Tumor-suppressor miRNAs, such as miR-101, miR-34a-5p, miR944, miR153-3p, miR126, miR-5580-3p, and miR376c-3p are often downregulated, whereas miR142-5p, miR-24, and miR-211 are upregulated and exhibits oncogenic properties.

miRNAs and OSCC						
Classes of miRNAs	Expression	Target	Phenotype	Role	Experimental model	Reference
miR-101	downregulated	*CXCR7*	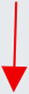 • cell adhesion • Survival • Tumor groth	Tumor suppressor	CAL27,Fadu,SCC-9 and SCC-27 human OSCC cell lines paraffin-embedded and fresh frozen tissue biopsies	(75)
miR-34a-5p	downregulated	*AXL*	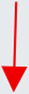 • EMT • invasion	Tumor suppressor	primary human fibroblast CAL27 and SCC15 human OSCC cell line xenograft OSCC mouse model	(76)
miR-944	downregulated	*MMP10*	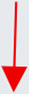 • migration • Invasion	Tumor suppressor	fresch frozen and FFPE tongue tumor samples AW13516, AW8507, CAL27 togue cancer cell lines and xenograft togue tumor mouse model	(77)
miR-153-3p	downregulated	*Snail*	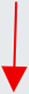 • EMT • invasion	Tumor suppressor	• human OSCC cell line SCC4	(78)
miR-126	downregulated	*VEGF-A*	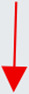 • EMT • invasion	Tumor suppressor	human OSCC cell lines, HSC3 and HSC4 cell FFPE human OSCC samples	(79)
miR-5580-3p	downregulated	*LAMC2*	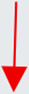 • survival • proliferation • migration	Tumor suppressor	SCC-4,SCC-9 and CAL 27 human OSCC cell lines FFPE human OSCC samples	(80)
miR376c-3p	downregulated	*HOXB7*	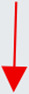 • survival • proliferation • migration • invasion	Tumor suppressor	SCC-4,SCC-9,SCC-15,SCC-25 human OSCC cell lines FFPE human OSCC samples	(81)
miR-142-5p	downregulated	*PTEN*	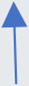 • invasion • survival • chemoresistance	oncogene	SAS and HSC-ME human OSCC cell lines	(82)
miR-24	downregulated	*PTEN*	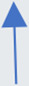 • invasion • survival • chemoresistance	oncogene	TSCC human OSCC samples	(83)
miR-211	downregulated	*BIN1*	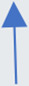 • proliferation • invasion • migration	oncogene	SCC6,SCC9,SCC25,HN4, and human PSCC cell lines FFPE human OSCC samples	(84)

Finally, several studies demonstrated that miRNAs could potentially serve as novel therapeutic tool for OSCC by modulating the basal expression. For instance, an upregulation of miR-375 significantly dampens cell proliferation, while inducing cell cycle arrest in the Gap 0/Gap 1 (G0/G1) phase of OSCC cells (61). Analogously, the overexpression of miR-148a in CAFs significantly altered the migration and invasion aptitude of OSCC cells by directly targeting *Wnt family member 10B* (*WNT10B*) (85). Restoring the intracellular levels of miR-1254, miR377, and miR-23a-3p suppressed cell proliferation and promoted apoptosis in OSCC (86–88).

### 4.2 CircRNAs: a promising target for the treatment of OSCC

Like miRNAs, circRNAs are also actively involved in the regulation of OSCC by altering pathways that underline cellular proliferation, migration, and invasion. In recent years, several circRNAs have been proposed to support both anti-tumoral and pro-tumoral programs using of various transcriptomic assays. In particular, the combination of RNA-seq and microarrays has enabled the identification of aberrant pathways associated with dysregulated circRNA expression. By serving as miR-876-5p sponge, circCDR1as (Cerebellar Degeneration-Related Protein 1 antisense) upregulates the expression level of *Solute Carrier Family 7 Member 11* (*SLC7A11*), which mediates OSCC progression (89). Moreover, OSCC exhibits an upregulation of circ_0001162 (also known as circular RNA derived from *matrix metalloproteinase-9* or *MMP-9*, circMMP-9), which, under physiological conditions, functions as a sponge for miR-149 via *AU-binding factor 1* (*AUF*) interaction, thus contributing to a more pronounced anti-tumoral phenotype (90). CircHIPK3 (Homeodomain Interacting Protein Kinase 3) sponges miR-381-3p and promotes OSCC progression through the regulation of Yes-associated protein 1 (YAP1) expression (91). The circFOXO3 (*Forkhead Box O3* or)/miR-214 axis enhances *Lysine Demethylase 2A* (*KDM2A*) levels, thereby sustaining metastatic spread (92). Li and colleagues have recently shed light on the role of circ_0000745 in promoting the progression of OSCC by regulating the expression of *cyclin D1* (*CCND1*) acting as a miR-488 sponge and partly through its binding with human antigen R (HuR) (93). CircRNAs also play a role in immune evasion mechanisms. Yang and colleagues demonstrated that circ*KRT1* [making keratin 1 (K1) protein] drives immune escape by sponging miR-495-3p, which ultimately regulates PD-L1 expression in OSCC samples (94). CircUHRF1 (circular RNA derived from the *UHRF1* gene) acts as a molecular sponge for miR-526b, leading to the upregulation of *Cellular Myelocytomatosis oncogene (MYC oncogene* or *c-Myc*). The increased *c-Myc* levels subsequently induce the transcription of EMT-promoting proteins, including *Epithelial Splicing Regulatory Protein 1* (*ESRP1)* and *Transforming Growth Factor Beta 1* (*TGFB1*) that provides instructions for the TGFβ-1 protein production (68). Recently, Hei and colleagues demonstrated that the development and progression of OSCC is promoted by the upregulation of circ_0020377, which acts as a sponge for miR-194-5p, thereby enhancing the expression of *Krüppel-like factor 7* (*KLF7)* and facilitating the activation of the EMT program in malignant cells (95). On the contrary, few evidence classifying circRNAs as anti-tumorigenic agents have been reported so far. *In vitro* and *in vivo* experiments revealed that circPKD2 (circular RNA derived from the *polycystic kidney disease 2* gene) can directly bind to miR-204, thus suppressing OSCC carcinogenesis through modulation of the *APC2*/ERK1/2/AKT*/*β-catenin signaling axis (71). The downregulated hsa_circ_0007059 was shown to simultaneously suppress the expression of *B-cell lymphoma* 2 (*Bcl-2*), *MMP-9*, and *CCND1*, while activating the AKT*/*mTOR pathway, and ultimately inhibiting cell proliferation, migration, and invasion (72). In addition, circSPATA6 (Spermatogenesis-associated protein 6) performs anti-oncogenic tasks by rewiring miR-182/*Tumor Necrosis Factor Receptor-associated Factor 6* (*TRAF6*) axis (96), Table 2.

**Table 2:** Involvement of different classes of circRNAs in the pathogenesis of OSCC. Several oncogenic (upregulated) and tumor suppressor circRNAs (downregulated) have been found to be involved in OSCC development and progression.

circRNAs and OSCC						
Classes of circRNAs	Expression	Target	Phenotype	Role	Experimental model	Reference
circCDR1as	upregulated	*SLC7A11*	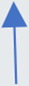 • survival	oncogene	CAL27 and SCC-9 human OSCC cell lines circCDR1 KD xenograft mouse model	(89)
circMMP-9	upregulated	*AUF1*	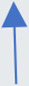 • EMT • invasion	oncogene	OSCC tissue samples HN4,UM1,SCC-9, SCC-15,HSC-3 and CAL 27 cell lines xenograft OSCC mouse model	(90)
circHIPK3	upregulated	*YAP1*	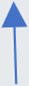 • progresion	oncogene	H357,SCC15,SCC-4 and SCC-9 human OSCC cell lines xenograft OSCC mouse model	(91)
circFOXO3	upregulated	*KDM2A*	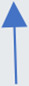 • invasion	oncogene	FaDu, CAL 27, UM1,UM2,SCC-4, SCC-9,SCC-25 OSCC cell lines frozen tissue samples xenograft OSCC mouse model	(92)
circ_0000745	upregulated	*CCND1*	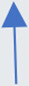 • progresion	oncogene	CAL 27, SCC-25, SCC9 and HSC-3 OSCC cell lines frozen tissue samples xenograft OSCC mouse model	(93)
circKRT1	upregulated	*PD-L1*	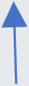 • Progresion • Immune invasion	oncogene	SCC-4,SCC-9,CAL27 and HSC-3 human OSCC cell lines FFPE human OSCC samples xenograft OSCC mouse model	(94)
circUHRF1	upregulated	*C_Myc* *ESRP1* *TGF-β1*	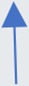 • proliferation • migration	oncogene	SCC25,CAL27,SCC15,and TSCCA human OSCC cell lines FFPE human OSCC samples xenograft OSCC mouse model	(68)
circ_0020377	upregulated	*KLF7*	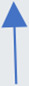 • proliferation • migration	oncogene	SCC-9 and HSC-3 human OSCC cell lines frozen tissue samples xenograft OSSC mouse model	(95)
circ_PDK2	upregulated	*APC2* *ERK ½* *AKT* *Β-catenin*	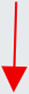 • proliferation • migration • invasion	tumor suppressor	SCC-15 human OSCC cell lines frozen tissue samples xenograft OSCC mouse model	(71)
circ_0007059	upregulated	*Bcl-2* *MMP-9* *CCND1* *mTOR*	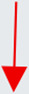 • proliferation • migration • invasion	tumor suppressor	HSCC9,SCC15,SCC25, and CAL27 OSCC cell lines frozen tissue samples xenograft OSCC mouse model	(72)
Circ_SPAT6	upregulated	*TRAF6*	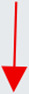 • proliferation • migration • invasion	tumor suppressor	CAL27 and SCC-9 human OSCC cell lines serum OSCC human samples xenograft OSCC mouse model	(96)

Therapies based on circular RNAs (circRNAs), which can function as microRNA (miRNA) sponges, are gaining significant interest, particularly when delivered via exosomes or lipid nanoparticles (LNPs). For LNP-based systems, a close correlation exists between lipid concentration and encapsulation efficiency. Optimizing this relationship is critical; for example, a common experimental approach involves testing various lipid concentrations while keeping the RNA concentration constant.

The versatility of these delivery systems stems from two key factors: the ease with which circRNAs are internalized by various cell types and the potential for precise targeting of affected sites (97). However, significant challenges remain. A primary limitation is the premature release of the therapeutic cargo before the nanoparticle reaches its target tumour.

While the development of nanomedicines is governed by strict regulations to ensure the safety and efficacy of clinically approved applications, the long-term safety of novel nanoparticle formulations requires further comprehensive investigation. Moreover, the clinical feasibility of using circRNAs in oral squamous cell carcinoma (OSCC) still requires validation with robust detection methods in large patient cohorts. Ultimately, more research is essential to explore the circRNA-miRNA networks that regulate the signaling pathways involved in the development and progression of oral cancer.

## 5 miRNAs and lncRNAs as potential candidates therapy against cancer metastasis

MiRNAs are emerging as promising candidates for anti-metastatic therapy. Two miRNA-based approaches are generally used. Anti-oncomiR therapy is used to silence the function of overexpressed oncogenic miRNAs, while miRNA mimics are used to restore the function of downregulated miRNAs (98, 99).

The activity of oncomiRs can be inhibited by the administration of synthetic anti-miRNA oligonucleotides (AMOs), locked nucleotide anti-miRNAs (LNAs), and miRNA sponges. These anti-miRNAs sequester or degrade mature miRNAs or compete with the target messenger for binding to the miRNA. Numerous potential miRNAs have been identified. For example, miR96 and miR-182 are overexpressed in most tumors and promote tumor cell migration and invasiveness, making them potential targets for inhibiting metastasis (100, 101). The same type of approach could be applied to lncRNAs. The use of lncRNAs as pharmacological targets has several advantages over other therapeutic strategies. LncRNAs are linear, small molecules and are therefore more druggable than proteins; their targeting, in fact, is mainly based on the sequence complementarity between nucleic acids (102). Their design would therefore be simpler, and their synthesis less expensive. The depletion of oncogenic lncRNAs could be achieved using siRNA or shRNA (small hairpin RNA). These molecules show great RNA selectivity and high knockdown efficiency (103).

However, in recent years, new methods have been developed that are replacing the RNA interference (RNAi) approach (i.e., antisense oligonucleotides, and aptamers). Antisense oligonucleotides (ASOs) are single-stranded DNA or RNA molecules with a sequence complementary to their target RNAs. When they bind target RNAs, RNase H1 recognizes the DNA:RNA/RNA:RNA heteroduplex and catalyzes the cleavage of the RNA molecules (104). Aptamers are small DNA, RNA, or peptide oligonucleotides with a stable three-dimensional structure that bind specifically to their targets (i.e., RNA, proteins, and small molecules), adapting to their three-dimensional shape; their targeting mechanism is therefore independent of sequence complementarity (105).

A deeper understanding of the molecular targets of lncRNAs, together with the development of new selective methods for the delivery of RNAs to tumor cells, could lead to significant benefits for the treatment of metastatic tumors. The high number of putatively druggable lncRNAs and therapeutic strategies based on lncRNAs could therefore prove to be very promising tools to complement the therapeutic strategies currently in use (106).

Thus, the spread of cancer is a challenging condition to manage, primarily due to its systemic diffusion locations and frequent mechanisms of chemotherapy resistance to therapeutic regimens, as well as the variable responses to anticancer drugs.

## 6 Conclusion and future perspectives

Despite the vast amount of data deriving from the integration of different multi-omics platforms that has revolutionized our understanding of OSCC biology, this neoplasm remains significantly challenging nowadays due to its etiology, late diagnosis, and resistance to conventional therapies. Integromics offers holistic view of the molecular mechanisms underpinning tumor onset and therapeutic response. Within this network, salivary miRNAs and circRNAs have emerged pivotal modulators of the expression of genes involved in different steps of tumorigenesis, including proliferation, angiogenesis, immune evasion, metastatic spread, and drug resistance. Thus, integrated multi-omics analysis of miRNA and circRNA signatures may guide the development of novel, non-invasive, diagnostic and prognostic tools, parallelly to the therapeutic potential of functional studies. Over the past decade, although cargo systems, such as exosomes and nanoparticles, have demonstrated pronounced attitude in attenuating cancer progression, thereby offering a promising frontier for RNA-based precision therapies, their clinical application still requires further validation. Looking ahead, AI-implemented integromics methodologies, at single cell or population level, will ultimately facilitate the development of patient-tailored interventions and improve clinical outcomes in OSCC.

## Glossary

3C: chromosome conformation capture
A3A: catalytic polypeptide-like 3A gene
ADH1B: alcohol dehydrogenase 1B
AI: artificial intelligence
AKR1C3: aldo-keto reductase family 1 member C3
AKT: protein kinase B
APC2: adenomatous polyposis coli protein 2
APOBEC3A: apolipoprotein B mRNA editing enzyme, catalytic polypeptide-like 3A
ATAC-Seq: assay for transposase-accessible chromatin using sequencing
AUF: AU-binding factor 1
AUX: AU-binding factor 1
AXL: tyrosine-protein kinase receptor UFO
Bcl-2: B-cell lymphoma 2
BIN1: bridging integrator-1
BisChIP-Seq: bisulfite-treated ChIP DNA-sequencing
*C. elegans*: *Caenorhabditis elegans*
CAF: cancer-associated fibroblast
CCND1: cyclin D1
CD274: programmed death-ligand 1gene
VEGFC: vascular endothelial growth factor C gene
CDH1: E-cadherin
CDR1as: cerebellar degeneration-related protein 1 antisense
ChIA-PET: chromatin interaction analysis with paired-end tag sequencing
ChIP-BMS: ChIP-bisulfite methylation sequencing
ChIRP: chromatin isolation by RNA purification
CHART: capture hybridization analysis of RNA targets
CircRNA: circular RNA
c-Myc: cellular myelocytomatosis oncogene
MYC oncogene: cellular myelocytomatosis oncogene
CTC: circulating tumor cells
ctDNA: circulating tumor DNA
CTLA4: cytotoxic T-lymphocyte associated protein 4
CXCR7: C-X-C chemokine receptor 7
CXCL12: C-X-C chemokine ligand 12
DART-Seq: deamination adjacent to RNA modification targets
DNase I: deoxyribonuclease I
DNase-Seq: DNase I hypersensitive site sequencing
EGFR: epidermal growth factor receptor
EMT: epithelial-to-mesenchymal
ERK: extracellular signal-regulated kinase
ESRP1: epithelial splicing regulatory protein 1
FFPE: formalin-fixed paraffin-embedded
FOXO3: forkhead box O3
G0/G1: gap 0/gap 1
GPR29: G-protein coupled receptor 29
HIPK3: homeodomain interacting protein kinase 3
HITS-CLIP: high-throughput sequencing of RNA isolated by crosslinking immunoprecipitation
HOXB7: homeobox B7
HuR: human antigen R
ISL1: insulin gene enhancer protein
KD: knockdown
KDM2A: lysine demethylase 2A
KLF7: krüppel-like factor 7
KRT1: keratin 1
LAMC2: laminin subunit gamma 2
LARP4: large tumor suppressor kinase
LATS2: large tumor suppressor kinase 2
LIGR-Seq: ligation of interacting RNA followed by high-throughput sequencing
m^6^A: N^6^-methyladenosine
m^6^A-LAIC-Seq: m^6^A-level and isoform-characterization sequencing
m^6^A-REF-Seq: m^6^A-sensitive RNA endoribonuclease–facilitated sequencing or m^6^A-REF-sequencing
m^6^A-SEAL: m^6^A-selective chemical labeling
SPATA6: spermatogenesis-associated protein 6
MAPK: mitogen-activated protein kinase
Methyl-HiC: methyl-high-throughput chromosome conformation capture
MeRIP-Seq: m^6^A/methylated RNA immunoprecipitation sequencing
MiRNA: microRNAs
MiCLIP: m^6^A individual-nucleotide resolution UV crosslinking and immunoprecipitation
MFAP4: microfibril associated protein 4
MMP-9: matrix metalloproteinase-9
MMP-10: matrix metalloproteinase-10
MNase-Seq: micrococcal nuclease sequencing
mRNA: messenger RNA
MS: mass spectrometry
mTOR: mammalian target of rapamycin
NcRNA: non-coding RNAs
NGS: next-generation sequencing
NMR: nuclear magnetic resonance
OSCC: oral squamous cell carcinoma
PCHi-C: promoter capture Hi-C
PD-L1: programmed death-ligand 1
PDCDL1: programmed death-ligand 1
PEA: proximity extension assay
PI3K: phosphatidylinositol 3-kinase
PKD2: polycystic kidney disease 2
PLA2G2A: phospholipase A2 group IIA
PTEN: phosphatase and tensin homolog deleted on chromo-some 10
RAP: RNA antisense purification
RAS: rat sarcoma
RIP-Seq: RNA immunoprecipitation sequencing
RISC: RNA-induced silencing complex
RNA-Seq: RNA sequencing
RRAS: RAS-related protein R-Ras
SAGE: serial analysis of gene expression
SC: single cell
ScRNA: single cell RNA sequencing
SMPS: single molecule protein sequencing
SLC7A11: solute carrier family 7 Member 11
Snail: zinc finger protein SNAI1
TCGA: cancer genome atlas
TGF-β1: transforming growth factor beta 1
TILLING: target induced local lesions in genomes
TNF: tumor necrosis factor
TNFSF11: tumor necrosis factor ligand superfamily member 11
TME: tumor microenvironment
TNM: tumor/nodes/metastasis
TRAF6: tumor necrosis factor receptor-associated Factor 6
TSCC: tongue squamous cell carcinoma
UHRF1: ubiquitin-like with PHD and RING finger domains 1
UTR: untranslated regions
VEGF-A: vascular endothelial growth factor A
VEGF-C: vascular endothelial growth factor C
WES: whole-exome sequencing
WGS: whole-genome sequencing
WHO: world health organization
WNT10B: Wnt family member 10B
YAP1: yes-associated protein 1.
